# Sarcopenia and Postoperative Outcomes Following Total Knee Arthroplasty: A Systematic Review of Observational Studies

**DOI:** 10.3390/jcm15145523

**Published:** 2026-07-14

**Authors:** Pierangelo Za, Marco Minelli, Carlo Esposito, Vincenzo Longobardi, Sebastiano Vasta, Giuseppe Calafiore, Federico Della Rocca

**Affiliations:** 1 IRCCS Ospedale San Raffaele, 20132 Milan, Italy; za.pierangelo@gmail.com (P.Z.);; 2 Department of Biomedical Sciences, Humanitas University, Via Rita Levi Montalcini 4, Pieve Emanuele, 20072 Milan, Italy; marcomariaminelli@gmail.com; 3 IRCCS Humanitas Research Hospital, Via Manzoni 56, Rozzano, 20089 Milan, Italyfederico.della_rocca@humanitas.it (F.D.R.); 4 Department of Movement, Human and Health Sciences, Foro Italico University of Rome, 00135 Rome, Italy; 5 Operative Research Unit of Orthopaedic and Trauma Surgery, Fondazione Policlinico Universitario Campus Bio-Medico, Via Álvaro del Portillo, 00128 Rome, Italy; 6 Research Unit of Orthopaedic and Trauma Surgery, Department of Medicine and Surgery, Università Campus Bio-Medico di Roma, Via Álvaro del Portillo, 00128 Rome, Italy; 7 Department of Orthopaedic and Trauma Surgery, Città di Parma Clinic, Piazzale Athos Maestri 5, 43123 Parma, Italy

**Keywords:** sarcopenia, total knee arthroplasty (TKA), postoperative complications, functional recovery, healthcare costs

## Abstract

**Purpose:** Sarcopenia has emerged as a potential prognostic factor for postoperative complications, functional recovery, patient-reported outcomes, and healthcare costs in patients undergoing total knee arthroplasty (TKA). This systematic review aimed to evaluate its impact on these outcomes following primary TKA. **Methods:** A systematic review was conducted according to PRISMA guidelines and prospectively registered in PROSPERO (CRD420261320013). PubMed/MEDLINE, EMBASE, and Cochrane Library were searched up to 1 December 2025. Comparative clinical studies including sarcopenic and non-sarcopenic patients undergoing primary TKA and reporting postoperative outcomes were included. Methodological quality was assessed using the MINORS tool. Due to heterogeneity in study design, diagnostic criteria, and outcome measures, a narrative synthesis was performed. **Results:** Nine studies including more than 93,000 patients were analyzed. Sarcopenia prevalence ranged from 7.7% to 25%. Sarcopenic patients demonstrated higher rates of postoperative complications, including medical events, blood transfusion, falls, fractures, reoperations, and implant-related complications. Functional recovery was delayed, particularly in patients with sarcopenic obesity, with slower improvements in range of motion and gait speed. Although both groups improved after TKA, short- to mid-term patient-reported outcomes were often inferior in sarcopenic patients, while long-term differences were less consistent. Sarcopenia was also associated with longer hospital stay and increased healthcare costs. **Conclusions:** Sarcopenia is associated with worse postoperative outcomes following primary TKA and may represent a modifiable risk factor for perioperative optimization.

## 1. Introduction

Sarcopenia is a multifactorial condition characterized by the progressive loss of skeletal muscle mass and strength [1,2]. According to the European Working Group on Sarcopenia in Older People (EWGSOP2), sarcopenia is defined as a muscle disease rooted in adverse muscle changes, where low muscle strength is the primary parameter, with low muscle quantity or quality confirming the diagnosis and poor physical performance indicating severity [3]. Clinically, this results in a gradual decline in muscle function [2]. This is associated with several adverse outcomes, including falls and fractures, disability, and increased mortality, particularly among older adults and hospitalized patients [4,5,6,7]. The reported prevalence of sarcopenia in the general population varies widely, ranging from 1% to 29% [8].

Notably, sarcopenia is frequently observed in patients with knee osteoarthritis who are candidates for total knee arthroplasty (TKA) [9,10]. A recent systematic review and meta-analysis estimated a pooled prevalence of 20.1% (95% CI 13.6–28.8%) among patients undergoing TKA, suggesting that approximately one in five TKA candidates may present with sarcopenia [11]. Knee osteoarthritis could further exacerbate sarcopenia, primarily through pain-related inactivity and reduced physical function [12]. Moreover, neuromodulatory mechanisms such as arthrogenic muscle inhibition (AMI) may further aggravate muscle weakness by impairing voluntary activation of periarticular musculature [9,13,14]. In this scenario, the coexistence of sarcopenia and the physiological stress of surgery may predispose patients to worse postoperative functional recovery, inferior clinical outcomes, and increased healthcare costs [15]. Collectively, these mechanisms may contribute to reduced postoperative quality of life and lower patient satisfaction following TKA [16,17].

In patients undergoing TKA, the identification of sarcopenia could be useful as part of a comprehensive preoperative assessment [16,17]. Given that approximately 10% of patients report dissatisfaction following TKA [18], the identification of modifiable risk factors remains essential to further improve outcomes. However, the available evidence regarding the association between sarcopenia and outcomes after TKA remains heterogeneous, with inconsistent findings across studies in terms of postoperative complications, functional recovery, patient-reported outcomes, length of stay, and healthcare costs. Therefore, a comprehensive synthesis of the literature is needed to clarify the current evidence and identify areas requiring further investigation. Accordingly, this systematic review aims to evaluate the association between sarcopenia and postoperative outcomes following TKA, specifically focusing on postoperative complications, functional recovery, patient-reported outcomes, length of hospital stay, and healthcare costs.

## 2. Materials and Methods

This systematic review was conducted in accordance with the Preferred Reporting Items for Systematic Reviews and Meta-Analyses (PRISMA) guidelines [19]. The review protocol was prospectively registered in the International Prospective Register of Systematic Reviews (PROSPERO) (registration number: CRD420261320013). The research question was formulated using the Population, Intervention, Comparison, and Outcomes (PICO) framework [20] as follows:

Population (P): patients with a diagnosis of sarcopenia and concomitant knee osteoarthritis;
Intervention (I): primary total knee arthroplasty (TKA);
Comparison (C): patients without sarcopenia undergoing primary TKA;
Outcomes (O): postoperative outcomes, including clinical and functional outcomes, patient-reported or quality-of-life measures, and complication rates.

The PRISMA 2020 checklist is provided in the Supplementary Materials (Table S1).

### 2.1. Literature Search

A comprehensive literature search was performed in PubMed/MEDLINE, EMBASE, and the Cochrane Library to identify all relevant studies evaluating the influence of sarcopenia on postoperative outcomes following TKA, in accordance with the predefined PICO framework. The search strategy combined Medical Subject Headings (MeSH) terms and free-text keywords as follows: (“Arthroplasty, Replacement, Knee”[Mesh] OR “total knee arthroplasty” OR “total knee replacement” OR TKA OR TKR) AND (“Sarcopenia”[Mesh] OR sarcopenia OR “muscle loss” OR “muscle wasting” OR “low muscle mass” OR “reduced muscle mass” OR “muscle weakness” OR frailty OR “age-related muscle loss”) AND (“clinical outcome” OR “functional outcome” OR “patient-reported outcome” OR PROM OR “quality of life” OR complication* OR revision OR failure OR mortality OR recovery OR satisfaction).

The final search was completed on 1 December 2025. In addition, the reference lists of included studies and relevant review articles were manually screened to minimize the risk of missing eligible publications.

### 2.2. Eligibility Criteria

Full-text studies were considered eligible for inclusion if they met the following criteria: (1) original comparative clinical studies; (2) inclusion of patients diagnosed with sarcopenia undergoing primary TKA; and (3) reporting of at least one postoperative outcome, including clinical outcomes, functional outcomes, patient-reported outcome measures (PROMs), quality-of-life measures, or complication rates.

Sarcopenia was defined as a muscle disorder characterized by reduced muscle strength, with low muscle quantity or quality used to confirm the diagnosis and impaired physical performance indicating severity. Studies using established diagnostic criteria, including the Asian Working Group for Sarcopenia (AWGS), the European Working Group on Sarcopenia in Older People (EWGSOP), or the Foundation for the National Institutes of Health (FNIH), as well as administrative definitions (e.g., ICD-9 coding), were considered eligible.

Exclusion criteria were revision TKA, animal or cadaveric studies, non-comparative studies, narrative or systematic reviews, editorials, and conference abstracts. Only studies published in English were included. No restrictions were applied regarding the year of publication.

### 2.3. Study Selection and Data Extraction

Two independent reviewers (P.Z and M.M) screened all retrieved records according to the predefined eligibility criteria. Titles and abstracts were initially reviewed, followed by full-text assessment of potentially eligible studies. A calibration exercise was conducted prior to screening to ensure consistency between reviewers, with inter-rater agreement assessed using Cohen’s kappa coefficient, indicating substantial agreement (κ = 0.86). Disagreements regarding study inclusion were resolved through discussion, and when consensus could not be reached, a senior reviewer adjudicated the final decision (G.C.).

Data extracted included first author, year of publication, study design, level of evidence, diagnostic criteria for sarcopenia, patient demographics (sex, age, body mass index), mean follow-up, postoperative complications, functional recovery, reported patient-reported outcome measures, length of stay and healthcare costs.

### 2.4. Risk of Bias Assessment

The methodological quality of the included studies was independently assessed by two reviewers (S.V. and C.E.) using the Methodological Index for Non-Randomized Studies (MINORS) tool. Any discrepancies in scoring were resolved through discussion, with adjudication by a senior author when necessary (F.D.R.).

### 2.5. Statistical Analysis

Given the heterogeneity among included studies in terms of study design, diagnostic criteria for sarcopenia, outcome measures, and follow-up duration, a quantitative meta-analysis was not performed. Therefore, a structured narrative synthesis was performed. Postoperative outcomes were summarized descriptively, and results were reported as presented in the original studies, including adjusted estimates when available. Differences in outcomes between sarcopenic and non-sarcopenic patients were qualitatively compared, with particular attention to consistency of findings across studies.

## 3. Results

### 3.1. Study Selection and Characteristics

**Figure 1:**
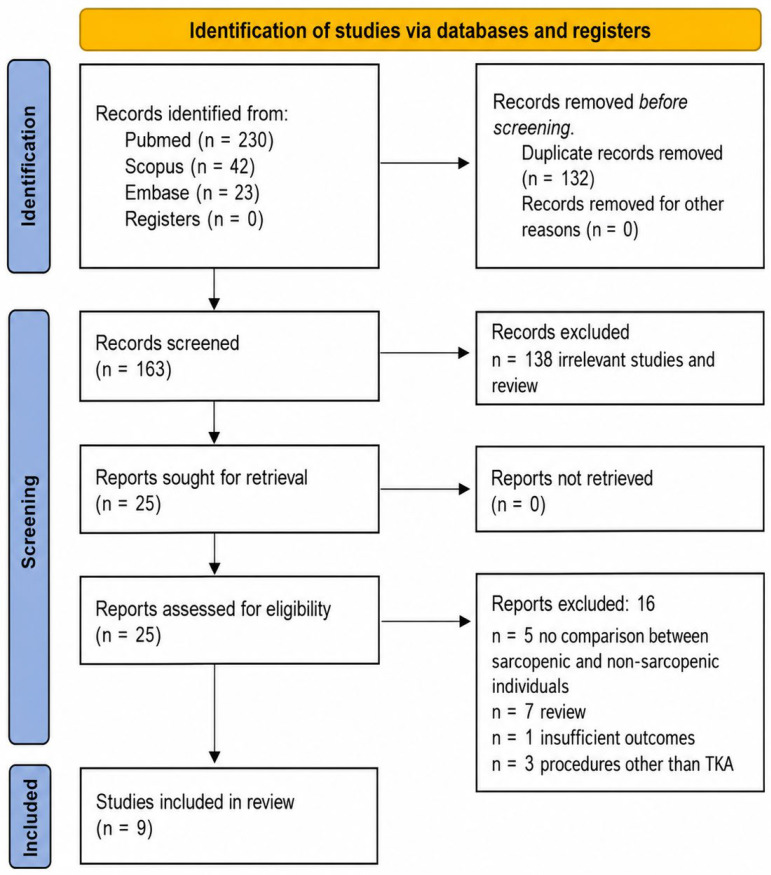
Flowchart of the selection process according to the PRISMA (Preferred Reporting Items for Systematic Reviews and Meta-Analyses) 2020 guidelines.

The study selection process is summarized in the PRISMA flow diagram (Figure 1). Nine studies were included in the qualitative synthesis [17,21,22,23,24,25,26,27,28]. A total of nine studies met the inclusion criteria, comprising seven retrospective cohort studies (level III evidence) [17,21,22,23,24,26,28] and two prospective observational studies (level II evidence) [25,27]. Overall, the studies were published between 2020 and 2024, and all included patients underwent primary total knee arthroplasty (TKA). The characteristics of the included studies are summarized in Table 1.

Across all included studies, more than 93,000 patients were evaluated, with sample sizes ranging from 20 to 90,438 participants. Mean patient age ranged from approximately 64.8 to 78.1 years, and sarcopenic patients were consistently older than non-sarcopenic controls. Body mass index (BMI) was generally lower in sarcopenic patients, except in studies focusing on sarcopenic obesity. Follow-up duration varied from 3 months to 60 months.

### 3.2. Diagnostic Criteria

The prevalence of sarcopenia varied considerably depending on diagnostic criteria and population characteristics, ranging from 7.7% to 25%. Most studies applied the Asian Working Group for Sarcopenia (AWGS) criteria [29], including those by Liao et al. [23,24], Ho et al. [25], Hwang et al. [26], Shon et al. [17], and Zhou et al. [28]. Alternative definitions were also used: European Working Group on Sarcopenia in Older People (EWGSOP) criteria [3] were applied alongside AWGS by Liao et al. [24]; Foundation for the National Institutes of Health (FNIH) criteria [30] were used by Tzartza et al. [27]; ICD-9 diagnostic coding was employed in the large Medicare database study by Ardeljan et al. [21]; and bioelectrical impedance-based muscle mass-only definitions were used in earlier cohort studies and subgroup analyses [22].

### 3.3. Postoperative Complications

Several studies demonstrated a significant association between sarcopenia and increased postoperative complications. The largest database study (90,438 patients) [21] reported that sarcopenic patients had significantly higher rates of 90-day medical complications (2.91% vs. 1.05%; OR 2.83), including acute anemia, blood transfusion, acute kidney injury, pneumonia, and urinary tract infections. Additionally, sarcopenia was associated with higher risks of falls (OR 3.54), lower-extremity fractures (OR 5.54), reoperation (OR 1.87), and implant-related complications at 2 years (OR 1.80). Two independent cohort studies confirmed an increased risk of postoperative blood transfusion among sarcopenic patients. Hwang et al. [26] reported higher transfusion rates (28.6% vs. 12.2%; OR 2.87), with skeletal muscle index demonstrating good predictive value (AUC 0.797). Similarly, Shon et al. [17] observed significantly higher transfusion rates (*p* < 0.001) and a higher incidence of periprosthetic joint infection (PJI) (*p* < 0.001) in sarcopenic patients. Zhou et al. [28] found that sarcopenic patients had higher surgical complication rates (26.2% vs. 7.1%, *p* = 0.019), particularly wound leakage (*p* = 0.021) and limb edema (*p* = 0.048), as well as longer length of stay (3.69 vs. 3.28 days, *p* = 0.038) and increased hospitalization costs (*p* = 0.015), although no differences in medical complications or 30-day readmission were observed.

### 3.4. Functional Recovery

Four studies evaluated postoperative functional recovery. Liao et al. [22] demonstrated that sarcopenic obesity was associated with delayed recovery of knee flexion range of motion (ROM), with a longer time to achieve ≥125° flexion (28 vs. 15 weeks) and a higher adjusted hazard ratio for poor ROM recovery (HR 1.68). In a subsequent study [23], sarcopenic obesity was associated with the longest time to recover gait speed ≥1.0 m/s (median 25 weeks vs. 12 weeks in normal patients), representing the highest risk of postoperative walking disability (adjusted HR 3.86). Sarcopenia alone (adjusted HR 2.75, *p* < 0.001) and obesity alone (adjusted HR 2.32, *p* < 0.001) were also independent risk factors for delayed functional recovery. Liao et al. [24] reported that sarcopenia severity was associated with significantly poorer improvements in timed up-and-go test, gait speed, timed chair rise, and Western Ontario and McMaster Universities Arthritis Index (WOMAC) physical function at 4 and 10 months postoperatively, compared with non-sarcopenic patients (all *p* < 0.05). Conversely, Tzartza et al. [27] did not find significant differences in short-term (3-month) Knee Injury and Osteoarthritis Outcome score (KOOS) improvement between sarcopenic and non-sarcopenic patients in a sample of 20 patients, despite worse baseline muscle mass in the sarcopenic group.

### 3.5. Patient-Reported Outcomes

Three studies specifically analyzed patient-reported outcome measures (PROMs). Short- to mid-term PROMs are inferior in sarcopenic patients, but long-term differences appear less consistent. Ho et al. [25] demonstrated that although both sarcopenic and non-sarcopenic patients improved significantly after TKA, in terms of WOMAC scores, Short-form-12-Health-Survey (SF-12) physical component scores, and gait speed, sarcopenic patients maintained significantly lower muscle mass indices at 12 months. Shon et al. [17] reported worse PROMs at 6 months in sarcopenic patients (all *p* < 0.001); however, differences were no longer significant at 12 months. Zhou et al. [28] found that patients in the sarcopenia group had significantly lower FJS-12 scores than those in the non-sarcopenia group (67.59 ± 9.81 vs. 72.47± 9.61, *p* = 0.024) at 60 months.

### 3.6. Length of Stay and Healthcare Costs

Two large retrospective cohort studies demonstrated that sarcopenia was associated with longer hospital length of stay and higher healthcare costs. The Medicare database analysis reported significantly longer hospitalization (4 vs. 3 days, *p* < 0.0001) and substantially higher day-of-surgery and 90-day episode-of-care costs in sarcopenic patients (all *p* < 0.0001) [21]. Similarly, Zhou et al. [28] confirmed longer length of stay (3.69 vs. 3.28 days, *p* = 0.038) and higher total hospitalization costs (*p* = 0.015) in the sarcopenic cohort.

### 3.7. Risk of Bias

The results of the methodological quality assessment using the Methodological Index for Non-Randomized Studies (MINORS) are summarized in Table 2. Overall, the included studies demonstrated moderate to low–moderate risk of bias, with total MINORS scores ranging from 16 to 21 out of a maximum of 24 points. Most studies were retrospective in design, with limited prospective data collection and no a priori sample size calculation, and blinding of outcome assessment was rarely reported. In addition, incomplete reporting of consecutive patient inclusion and follow-up completeness may have introduced selection and attrition bias.

## 4. Discussion

The present systematic review suggests that sarcopenia could be a clinically relevant prognostic factor in patients undergoing primary total knee arthroplasty (TKA). Across nine studies encompassing more than 93,000 patients, sarcopenia was associated with higher postoperative complication rates, delayed functional recovery, longer hospital length of stay, and increased healthcare costs. Although patient-reported outcome measures (PROMs) tended to improve after TKA regardless of sarcopenia status, short- to mid-term outcomes were often inferior in sarcopenic patients. An important consideration is whether sarcopenia represents an independent prognostic factor or rather a surrogate marker of broader frailty and physiological vulnerability. Sarcopenia frequently coexists with advanced age, comorbidities, and reduced functional reserve, all of which may independently influence postoperative outcomes [29]. Therefore, the observed associations may reflect the cumulative burden of frailty rather than the isolated effect of reduced muscle mass and strength.

One of the findings of this review is the association between sarcopenia and increased postoperative complications. Indeed, higher rates of medical complications, blood transfusion, falls, fractures, reoperations, and implant-related complications among sarcopenic patients were observed in database and cohort studies. These findings are consistent with a previous systematic review and meta-analysis, which found that sarcopenia was associated with a higher risk of overall complications following TKA (RR = 1.80, 95% CI: 1.53–2.11; *p* < 0.001) and substantially greater blood transfusion requirements (RR = 3.66, 95% CI: 2.86–4.69; *p* < 0.001) [30]. These associations may be interpreted in the context of the physiological characteristics underlying sarcopenia. Sarcopenia is defined by the AWGS criteria [31] as low muscle mass with reduced strength and/or physical performance, and by the EWGSOP and FNIH criteria [3,32] as a low muscle strength condition confirmed by low muscle quantity. Moreover, sarcopenia reflects a loss of physiological reserve and resilience, a process that is increasingly attributed to chronic low-grade inflammation, with pro-inflammatory cytokines such as IL-6 and TNF-α contributing to impaired muscle protein synthesis and enhanced proteolysis [33,34]. Thus, sarcopenia reflects reduced physiological reserve, impaired muscle-mediated joint protection, and altered metabolic and inflammatory responses, all of which may predispose patients to perioperative morbidity. Moreover, the higher risk of postoperative blood transfusion observed across multiple studies may be explained by lower baseline hemoglobin levels in sarcopenic patients, who have been shown to exhibit significantly lower hemoglobin compared with non-sarcopenic controls, as well as by reduced protein stores and impaired anabolic responses [35].

Functional recovery after TKA was negatively associated with sarcopenia, particularly when combined with obesity. The poorer outcomes observed in sarcopenic obesity could be secondary to the synergistic detrimental effects of excess fat mass and reduced muscle mass on postoperative recovery. These findings are consistent with evidence reported in a recent narrative review, which identified sarcopenia as an important factor associated with impaired early functional recovery and delayed ambulation with a greater reliance on assistive devices following TKA [36]. Studies evaluating rehabilitation outcomes reported delayed recovery of knee range of motion and gait speed in sarcopenic and sarcopenic-obese patients, along with inferior improvements in objective functional measures such as the timed up-and-go and chair rise tests. Lower skeletal muscle mass index has been shown to adversely affect rehabilitation outcomes after TKA, suggesting that greater muscle mass facilitates more effective postoperative functional recovery [23]. In addition, muscle weakness—especially of the quadriceps—is a key determinant of functional performance after TKA, with progressive postoperative strength loss correlating with delayed recovery of mobility and physical function [37]. Collectively, these findings suggest that sarcopenia is associated with impaired neuromuscular performance and reduced rehabilitation capacity, although it may also represent a surrogate marker of broader frailty and diminished physiological reserve.

With regard to PROMs, the evidence was more heterogeneous. While some studies reported worse early PROMs in sarcopenic patients, these differences tended to diminish by 12 months postoperatively. This may reflect the strong analgesic effect of TKA and the capacity of standardized rehabilitation protocols to partially mitigate the functional consequences of sarcopenia over time. Indeed, rehabilitation after TKA can improve muscle strength and muscle mass, which in turn is associated with better measures of functional performance such as sit-to-stand and gait tests [38]. Nevertheless, patient-reported outcome measures are known to exhibit ceiling and plateau effects after TKA and may fail to capture persistent deficits in muscle strength and objective functional performance, which can remain impaired despite favorable PROMs [39].

The economic implications of sarcopenia were also noteworthy. In a large systematic review and meta-analysis, the prevalence of sarcopenia in older adults was observed to vary between ~10% and 27% in the general population [40]. Since cohort and database studies reported longer hospital stays and higher day-of-surgery and 90-day episode-of-care costs in sarcopenic patients, these findings suggest the relevance of sarcopenia not only as a clinical risk factor but also as a determinant of healthcare resource utilization, particularly in the context of value-based care and bundled payment models.

The findings of this review may support the routine consideration of sarcopenia in the preoperative assessment of patients undergoing TKA. Given the observational nature of the included studies, the findings should be interpreted as associations rather than evidence of a causal relationship between sarcopenia and postoperative outcomes. Since sarcopenia was associated with increased complications, delayed recovery, and increased costs, it could represent a potentially modifiable risk factor. The preoperative period may represent a potential window for targeted interventions, including nutritional optimization, resistance training, and multimodal prehabilitation programs aimed at improving muscle mass and functional performance [41,42,43]. Indeed, a recent review reported that merged prehabilitation and dietary supplementation could improve the functional capacity and ability to withstand the upcoming surgical stressors in older people undergoing knee arthroplasty [41]. Identifying high-risk phenotypes, such as sarcopenic obesity, may further allow individualized perioperative strategies [41,42,43]. However, the effectiveness of strategies aimed at modifying sarcopenia to improve postoperative outcomes remains to be established.

A major challenge identified in this review is the substantial heterogeneity in sarcopenia definitions. Although most studies applied the Asian Working Group for Sarcopenia (AWGS) criteria, others used EWGSOP, FNIH criteria, ICD-9 diagnostic codes, or muscle mass-only definitions based on bioelectrical impedance analysis. This variability likely contributed to the wide range of reported sarcopenia prevalence (7.7% to 25%) and limits direct comparison across studies. Importantly, studies relying solely on muscle mass measurements may underestimate the functional dimension of sarcopenia, whereas coding-based definitions may lack diagnostic specificity. Standardization of diagnostic criteria in future research is therefore essential to improve comparability and strengthen the evidence base. Another relevant issue is the lack of consistent reporting of sarcopenia severity. According to EWGSOP2 [3], sarcopenia may be graded as probable sarcopenia when low muscle strength is present, confirmed sarcopenia when low muscle strength is accompanied by reduced muscle quantity or quality, and severe sarcopenia when impaired physical performance is also present. Similarly, AWGS 2019 [44] recognizes severe sarcopenia when low muscle mass, low muscle strength, and poor physical performance coexist. In the context of TKA, it is plausible that more advanced sarcopenia stages may be associated with greater perioperative vulnerability, poorer rehabilitation tolerance, and a higher risk of adverse outcomes. This is relevant from a clinical perspective: the identification of sarcopenia should not currently be interpreted as a contraindication to TKA, since no validated sarcopenia threshold has been established beyond which surgery should be avoided. Rather, sarcopenia should be considered a marker of increased perioperative vulnerability that may help guide risk stratification and optimization strategies. However, the available literature rarely stratifies patients according to sarcopenia severity, and the current evidence does not allow a reliable stage-specific risk assessment. Future studies should therefore report sarcopenia severity using standardized criteria to determine whether a dose–response relationship exists between sarcopenia stage and postoperative complications and functional recovery.

This review has several other limitations. First, most included studies were retrospective and observational, limiting causal inference and increasing the risk of residual confounding despite adjustment strategies. Moreover, sarcopenia may represent a surrogate marker of broader frailty and physiological vulnerability, and the extent to which it is independently associated with postoperative outcomes remains unclear. Second, in addition to the heterogeneity in sarcopenia definitions discussed above, variability in outcome measures and follow-up duration limited comparability across studies and precluded quantitative meta-analysis. The marked differences in sample size, ranging from small single-center cohorts to large administrative database studies, should also be considered when interpreting the overall findings. Third, differences in perioperative care, rehabilitation protocols, and healthcare systems were not consistently accounted for and may have contributed to outcome heterogeneity. In addition, the predominance of studies conducted in Asian populations may limit the generalizability of the findings to other populations and healthcare settings. Finally, long-term functional and patient-reported outcomes were infrequently reported, while objective performance-based measures were inconsistently assessed, limiting conclusions regarding the durability and clinical significance of the observed associations.

## 5. Conclusions

In conclusion, the available evidence suggests that sarcopenia is associated with worse postoperative outcomes following primary TKA, including higher complication rates, delayed functional recovery, longer hospital stays, and increased healthcare costs. Although patient-reported outcomes generally improve after surgery, sarcopenic patients may remain at a relative biological and functional disadvantage, particularly in the early postoperative period. Future prospective studies using standardized diagnostic criteria and objective functional measures are needed to clarify whether sarcopenia is independently associated with postoperative outcomes and to determine whether targeted multimodal prehabilitation strategies could modify these outcomes.

## Figures and Tables

**Table 1:** Summary of the included studies.

Author, Year	Type of Study	Level of Evidence	Patients Characteristics	Out Come of Interest	Methods	Sarcopenia Evaluation	Patients	Sex (M/F)	Mean FU (Months)	Mean Age	Mean BMI	Main Findings
Ardeljan et al. [20], 2022	Retrospective database cohort study	III	Patients who underwent primary TKA and sarcopenic patients were identified using ICD-9 procedural and diagnosis codes. Patients who underwent primary TKA without sarcopenia served as controls.	Primary outcomes: in-hospital length of stay, 90-day medical complications, falls, lower extremity fractures, reoperations, 2-year implant-related complications, and day-of-surgery and 90-day episode-of-care costs.	Retrospective review of the Medicare patient population database from 1 January 2005 to 31 March 2014 Study group patients were randomly matched to controls in a 1:5 ratio by age, gender, chronic obstructive pulmonary disease (COPD), diabetes mellitus, hyperlipidemia, hypertension, obesity, and tobacco use.	Identified by ICD-9 code 728.2; severity not assessable and diagnostic criteria not specified.	90,438 patients; sarcopenic: 15,073 (16.7%); non sarcopenic: 75,365	Sarcopenic group: 4527 M/10,546 F; non sarcopenic: 22,635 M/52,730 F	24	not applicable	not applicable	Sarcopenia associated with: - Longer hospital stay (4 vs. 3 days, *p* < 0.0001). - Higher costs, (9.2% higher day-of-surgery costs and 16.9% higher 90-day episode-of-care costs; all *p* < 0.0001). - 90-day medical complications (2.91% vs. 1.05%; OR 2.83), including acute anemia (OR 4.43), blood transfusion (OR 4.24), AKI (OR 2.33), pneumonia (OR 1.94) and UTI (OR 1.64). - Higher risk of 90-day falls (OR 3.54), lower-extremity fractures (OR 5.54) and reoperation (OR 1.87). - Increased implant-related complications (4.29% vs. 2.42%; OR 1.80).
Liao D. et al., Nutrients [21], 2021	Retrospective cohort study	III	≥ 60 years, K-L grade ≥3 who underwent primary unilateral TKA and had a knee flexion ROM ≥ 125 degrees at discharge were divided into three groups: Non-obesity; Obesity and Sarcopenic-Obesity	Effects of sarcopenic obesity on the knee flexion ROM outcome after TKA in older adults with KOA	BMI ≥ 25 kg/m^2^ to define obesity; Active knee flexion ROM was measured before surgery (baseline), at discharge and at outpatient follow-up admissions. After inpatient discharge, each patient was asked to attend outpatient follow-up clinic one to two times per month until a full recovery of knee flexion ROM (130–135°) was achieved.	AWGS 2019 criteria	587 knees: non-obesity (n = 205); obesity (n = 323); sarcopenic obesity (n = 59)	144 M/443 W	9	Non-obesity: 70.9 ± 6.9; Obesity: 67.8 ± 6.3; Sarcopenic-Obesity: 75.1 ± 6.1	NO group: 22.9 ± 1.8; O: 30.6 ± 3.9; SO: 27.0 ± 2.1	Sarcopenic obesity was associated with worse ROM recovery after TKA: higher risk of poor outcome (ROM < 125°) vs. non-obese (adjusted HR 1.68, 95% CI 1.06–2.66) and longer time to reach ROM ≥ 125° (28 vs. 15 weeks). Obesity alone also increased risk (adjusted HR 1.35, *p* < 0.05).
Liao D. et al., JAMDA [22], 2022	Retrospective cohort study	III	≥65 years with K–L grade ≥ 3 undergoing primary unilateral TKA; revision surgery and major systemic or neurologic diseases excluded. Patients with preserved gait speed (≥1.0 m/s) before surgery or at hospital discharge were excluded to ensure initial walking disability.	Association between body composition phenotypes (sarcopenia, obesity, sarcopenic obesity) and walking disability during rehabilitation after TKA, with focus on sarcopenic obesity as a high-risk profile.	Patient appendicular lean mass was measured using bioelectrical impedance analysis by an InBody 220 apparatus (Biospace Co.). Walking disability was assessed by gait speed; time to achieve gait speed ≥ 1.0 m/s during 8-month follow-up. Patients were classified into 4 groups: sarcopenic obese, sarcopenic, obese, and normal (reference group).	AWGS 2019 criteria	418 patients; normal reference: 170; sarcopenic: 122; obese: 132; and sarcopenic obese: 58.	normal: 49 M/131 F; sarcopenic: 34 M/88 F; obese: 31 M/101 F; sarcopenic-obese: 15 F/43 M	8	normal: 70.1± 5.1; sarcopenic: 76.1 ± 5.6; obese: 69.7 ± 3.9; sarcopenic-obese: 72.3 ± 3.5	normal: 25.2 ± 2.6; sarcopenic: 23.2 ± 2.3; obese: 33.5 ± 3.3; sarcopenic-obese: 31.1 ± 1.1	Sarcopenic obese patients had the longest recovery time to gait speed ≥ 1.0 m/s (median 25 weeks vs. 12 weeks in normal). Sarcopenic obesity was associated with the highest risk of postoperative walking disability (adjusted HR 3.86, 95% CI 2.65–5.64), followed by sarcopenia (HR 2.75) and obesity (HR 2.32).
Liao D. et al., Ther Adv Musculoskel Dis [23], 2021	Retrospective cohort study	III	Age between 50 and 85 years, undergoing primary unilateral TKA; major comorbidities and revision cases excluded.	Impact of preoperative sarcopenia severity (none vs. class I vs. class II) on postoperative rehabilitation outcomes after TKA in patients with knee OA, comparing rehabilitation treatment effects between groups.	Skeletal muscle index (SMI, kg/m^2^) measured by bioelectrical impedance analysis (InBody 220). Functional sarcopenia outcomes (timed up-and-go test (TUGT), gait speed, timed chair rise) and patient-reported outcomes (WOMAC pain, stiffness, function) assessed at baseline, 4 and 10 months after TKA.	AWGS 2019 definition: class I and II sarcopenia = 1 and 2 SD below young reference mean (men: 9.87/8.87 kg/m^2^; women: 7.15/6.42 kg/m^2^). EWGSOP definition also applied (men: 10.76/8.50 kg/m^2^; women: 6.76/5.75 kg/m^2^ for moderate/severe sarcopenia).	190 patients; According to AWGS: no sarcopenia n = 69, class I n = 65, class II n = 56. According to EWGSOP: no sarcopenia n = 86, class I n = 79, class II n = 25.	12 M/178 F	10	AWGS: 69.2 ± 6.6 (no), 73.0 ± 6.2 (class I), 76.4 ± 6.1 (class II). EWGSOP: 70.9 ± 6.5 (no), 72.7 ± 6.6 (class I), 78.1 ± 6.6 (class II).	AWGS: 24.7 ± 3.9 (no), 26.7 ± 3.0 (class I), 30.1 ± 3.7 (class II). EWGSOP: 25.6 ± 3.6 (no), 27.3 ± 3.9 (class I), 28.2 ± 4.2 (class II).	Sarcopenia prevalence varied by definition (AWGS: no 69, class I 65, class II 56; EWGSOP: no 86, class I 79, class II 25). Higher sarcopenia class was associated with older age, higher BMI and worse baseline function (*p* < 0.01). After adjustment, non-sarcopenic patients showed greater improvements than class II sarcopenic patients in TUGT (aMD −3.2 s), gait speed (+0.57 m/s), TCR (+2.9 repetitions) and WOMAC-PF (−11.2) at 4 and 10 months.
Ho et al. [25], 2021	Prospective observational study	II	≥50 years, end-stage knee OA who underwent TKA	Effect of sarcopenia on postoperative pain and functional outcomes after TKA (comparison between sarcopenic and non-sarcopenic patients).	DXA-based body composition (LMI, ALMI); sarcopenia defined by AWGS criteria (low muscle mass, grip strength, or gait speed). Muscle strength (handgrip, knee flexion/extension), physical performance (6 m gait speed), WOMAC, SF-12 (PCS/MCS), and IPAQ assessed at baseline, 6 and 12 months after TKA.	AWGS 2019 criteria	58 patients: sarcopenic group (19) vs. non-sarcopenic (39)	12 M/46 F	12	sarcopenic patients: 67.89 ± 7.07; non-sarcopenic: 67.92 ± 6.85	sarcopenic: 25.64 ± 2.64; non-sarcopenic: 28.57 ± 4.04	After TKA, muscle mass increased in both groups (LMI sarcopenic 12.93 vs. 13.27; non-sarcopenic 14.96 vs. 15.42), but ALMI and LMI remained significantly lower in sarcopenic patients at 12 months (ALMI 5.38 vs. 6.28; LMI 13.39 vs. 15.42; all *p* < 0.01).
Hwang et al. [26], 2022	Retrospective cohort study	III	Patients undergoing primary TKA for degenerative knee arthritis with preoperative bioelectrical impedance analysis (BIA) assessment; bilateral procedures, inadequate hydration status and severe obesity were excluded	Association between low muscle mass (BIA-defined sarcopenia) and postoperative blood transfusion, delirium, and acute kidney injury (AKI) after TKA.	Skeletal Muscle Index (SMI) (ASM/height^2^) measured by BIA. Preoperative and postoperative hemoglobin levels, occurrence of postoperative blood transfusion, delirium and acute kidney injury	AWGS 2019 criteria	452 patients, sarcopenic group: 35; non-sarcopenic: 417	60 M/392 F	none	Sarcopenic: 74.5 ± 6.5; non-sarcopenic: 70.5 ± 6.6 years	Sarcopenic: 23.9 ± 3.4; non-sarcopenic: 26.7 ± 3.2	Low muscle mass prevalence was 7.7% (35/452). Sarcopenic patients were older, had lower BMI (23.9 vs. 26.7 kg/m^2^), lower preoperative Hb (12.2 vs. 13.0 g/dL) and total protein (all *p* ≤ 0.004). Sarcopenia was associated with higher postoperative blood transfusion rates (28.6% vs. 12.2%; OR 2.87, 95% CI 1.25–6.17; *p* = 0.009).
Shon, O.-J. et al. [17], 2023	Retrospective cohort study	III	Age > 60 years, K–L ≥ 3 undergoing primary TKA. Excluded: rheumatoid arthritis, post-traumatic OA, non-independent ambulation.	Primary: prevalence of sarcopenia in patients undergoing TKA for end-stage knee OA. Secondary: effect of sarcopenia on PROMs and identification of predisposing factors.	Sarcopenia defined using: - Whole-body DXA, with calculation of height-adjusted skeletal muscle index (SMI) and appendicular skeletal muscle index (ASMI); - Handgrip strength: dynamometry (Southampton protocol; low strength < 28 kg men, <18 kg women). - 6 m gait speed (low performance < 1.0 m/s). Baseline demographics, comorbidities (mCCI), Hb and total protein assessed. PROMs evaluated by KOOS and WOMAC; knee ROM measured by goniometer.	AWGS 2019 criteria	445 patients; sarcopenic group: 42; non-sarcopenic: 403	52 M/393 F	12	Sarcopenic group: 76.2; non-sarcopenic: 67.8	Sarcopenic group: 23.2 ± 3.2; non-sarcopenic: 27.6 ± 3.1	Sarcopenia prevalence was 9.4% (higher in men: 15.4% vs. women: 8.7%) and increased with age (*p* < 0.001). Sarcopenia is associated with higher transfusion (*p* < 0.001) and PJI rates (*p* = 0.009) and worse short-term outcomes, with no differences at long-term follow-up. Independent risk factors for sarcopenia were older age (OR 1.4), lower BMI (OR 0.7) and higher mCCI (OR 1.2).
Tzartza, C.L. et al. [26], 2023	Prospective observational study	II	Patients with knee OA undergoing primary TKA at a single center	Impact of sarcopenia on postoperative symptom improvement and functional recovery after TKA in patients with knee osteoarthritis.	Muscle mass estimated by BIA (Quadscan 4000) and handgrip measured with Takei dynamometer. KOOS evaluated preoperatively and 3 months postoperatively to assess symptom and functional improvement.	Sarcopenia defined according to FNIH criteria. Cut-offs: handgrip < 26 kg (men), <16 kg (women); ALM < 19.75 kg (men), <15.02 kg (women); ALM/BMI < 0.789 (men), <0.512 (women).	20 patients; sarcopenic group: 5; non-sarcopenic: 15	9 M/11 F	3	Sarcopenic: 76.4 ± 8.05; non sarcopenic: 64.8 ± 10.51	Sarcopenic: 29.54 ± 6.20; Non-sarcopenic: 29.39 ± 4.18	Sarcopenia prevalence was 25% (5/20). Sarcopenic patients were older than controls (*p* = 0.038). No significant differences in clinical outcomes between sarcopenic and non-sarcopenic patients despite baseline differences.
Zhou et al. [28], 2024	Retrospective cohort study	III	Patients undergoing primary TKA for knee OA with available handgrip strength assessment and complete clinical and laboratory data; bilateral TKA excluded	Impact of sarcopenia on postoperative complications, length of stay, hospitalization costs and blood loss after TKA under ERAS protocol.	Baseline demographics, comorbidities (ASA, hypertension, DM, osteoporosis) and laboratory data (Hb, Hct, albumin) collected. Outcomes included medical and surgical complications, 30-day readmission and PJI; LOS, operative time, total hospitalization cost; estimated blood loss, transfusion rate and maximal Hb/Hct/Alb reduction; and patient-reported outcome (12-item forgotten joint score: FJS-12).	AWGS 2019 criteria	291 patients, 58 (19.9%) sarcopenic and 233 (80.1%) non-sarcopenic; after 1:1 propensity score matching, 84 patients: 42 sarcopenic matched with 42 non-sarcopenic	Sarcopenic group: 8 M/34 F; non-sarcopenic: 8 M/34 F	60	Sarcopenic: 69.57 ± 6.73; non-sarcopenic: 70.95± 5.51	not reported	Sarcopenia associated with: - Higher surgical complication rates (26.2% vs. 7.1%, *p* = 0.019), mainly wound leakage (*p* = 0.021) and limb edema (*p* = 0.048), - Longer LOS (3.69 vs. 3.28 days, *p* = 0.038); - Higher hospitalization costs (*p* = 0.015). - Worse FJS-12 scores (67.59 ± 9.81 vs. 72.47 ± 9.61, *p* = 0.024).

**Table 2:** Methodological quality assessment using the Methodological Index for Non-Randomized Studies (MINORS).

Study	Aim	Consecutive Patients	Prospective Data Collection	Appropriate Endpoints	Unbiased Assessment	Adequate Follow-Up	<5% Loss to Follow-Up	Sample Size Calculation	Adequate Control Group	Contemporary Groups	Baseline Equivalence	Adequate Statistics	Total (/24)	Risk of Bias
Ardeljan et al. [20]	2	1	0	2	1	2	2	0	2	2	2	2	18	Low-moderate
Liao D., Nutrients [21]	2	1	0	2	1	2	1	0	2	2	1	2	16	Moderate
Liao D, JAMDA [22]	2	1	0	2	2	2	1	0	2	2	1	2	17	Moderate
Liao D., Ther Adv Musculoskel Dis [23]	2	1	0	2	1	2	1	2	2	2	1	2	18	Low-moderate
Ho et al. [25]	2	2	2	2	1	2	1	2	2	2	1	2	21	Low-moderate
Hwang et al. [26]	2	1	0	2	1	2	2	2	2	2	2	2	20	Low-moderate
Shon, O-J. et al. [17]	2	2	0	2	1	2	2	0	2	2	1	2	18	Low-moderate
Zhou et al. [28]	2	2	0	2	2	2	2	0	2	2	2	2	20	Low-moderate
Tzartza, C.L. et al. [27]	2	1	2	2	1	1	2	0	1	2	1	1	16	Moderate

## Data Availability

No additional materials are publicly available. The data extracted from the included studies and the risk of bias assessments are fully reported within the tables of the manuscript. No analytic code was generated or used. Further details are available from the corresponding author upon reasonable request.

## Supplementary Materials

The following supporting information can be downloaded at: https://www.mdpi.com/article/10.3390/jcm15145523/s1, Table S1: PRISMA 2020 Checklist.
